# Artificial Chromosome Doubling in Allotetraploid *Calendula officinalis*

**DOI:** 10.3389/fpls.2020.00622

**Published:** 2020-05-29

**Authors:** Ghasem Esmaeili, Katrijn Van Laere, Hilde Muylle, Leen Leus

**Affiliations:** ^1^Department of Horticulture, Azadi Square, Faculty of Agriculture, Ferdowsi University of Mashhad, Mashhad, Iran; ^2^Flanders Research Institute for Agriculture, Fisheries and Food (ILVO), Plant Sciences Unit, Melle, Belgium

**Keywords:** colchicine, fluorescent *in situ* hybridization, FISH, oryzalin, polyploidy, trifluralin

## Abstract

*Calendula officinalis* L. is known as an ornamental plant as well as a source of biochemical compounds used in cosmetics and industry. *C. officinalis* has a complex karyotype. Published chromosome numbers differ between 2*n* = 4*x* = 28 or 32. We have estimated genome sizes in nine commercial cultivars and evaluated the ploidy level by karyotyping and fluorescent *in situ* hybridization (FISH) using 5S and 45S rDNA loci. The detection of chromosome sets of two rather than four homologues would suggest that *C. officinalis* has an allotetraploid background. In addition, four signals for 45S but only two for 5S were found by using FISH. Artificial chromosome doubling is a common technique in plant breeding, as polyploidization results in several consequences for plant growth and development. Especially the suggested allotetraploid background in *C. officinalis* is interesting when examining the effect of chromosome doubling on the plant phenotype. Here we describe chromosome doubling of three allotetraploid cultivars of *C. officinalis*, ‘Nova,’ ‘WUR 1553-7’ and ‘Orange Beauty’. Three antimitotic agents – colchicine, oryzalin and trifluralin - were used in different concentrations to find the combination of the best agent and the best dosage to obtain octaploids. For all three cultivars a few octaploids were obtained. A concentration of 200 and 400 ppm of colchicine was most efficient for chromosome doubling in ‘Nova’ and ‘Orange Beauty,’ respectively. For ‘WUR 1553-7’ the treatment with 20 ppm oryzalin was also effective. Cell numbers and first observations of the phenotype in the chromosome doubled plants show thicker leaves and bigger cells, as commonly observed after ploidy doubling. Due to the low number of chromosome doubled plants obtained more elaborate phenotyping will be performed on following generations cultivated under field conditions.

## Introduction

The genus *Calendula* is native to the Mediterranean region, Macronesia and Southwest Asia. It is a taxonomically complex genus, with 10–27 species being reported depending on the author. *C. officinalis* L. is an important annual medicinal and ornamental plant native to Europe and North Africa (Ao, 2007). Several classes of biochemical compounds were identified in *C. officinalis* inflorescences including essential oil, carotenoids, flavonoids, terpenoids, coumarins, quinones, amino acids, lipids, and carbohydrates. These compounds can be used for various pharmaceutical and medicinal purposes: as an anti-oxidant, anti-inflammatory, anti-bacterial, anti-fungal, anti-cancer, anti-HIV, to promote wound healing, and more (Khalid and da Silva, 2012; Jan and John, 2017; Cruceriu et al., 2018; Verma et al., 2018; Givol et al., 2019).

Interest in *C. officinalis* is growing as a good source of bio-compounds. It is known that within *C. officinalis* the content of biochemical compounds varies widely, depending on the color of the inflorescence and the rate of ligulate florets (Khalid and da Silva, 2012). The performance of existing cultivars is therefore being screened with the aim of selecting higher yielding cultivars. In addition, (artificial) polyploidization is a successful method to improve plant traits. Polyploidy has an effect on biosynthesis pathways and gene expression (Dhooghe et al., 2010; Sattler et al., 2016; Salma et al., 2017; Zhang et al., 2018). Polyploids may have bigger inflorescences, larger and thicker leaves and stems, larger pollen, a higher biomass, higher concentration of secondary metabolites, and better tolerance to environmental stress (Mori et al., 2016; Li et al., 2018; Luo et al., 2018; Eng and Ho, 2019). Polyploidy has led to an increase in secondary metabolites in medicinal plants in two ways: first, by the increase of biomass in chromosome doubled plants, and second, by the increase in number of gene copies related to the pathways of the secondary metabolites. Examples of higher levels of secondary metabolites after chromosome doubling are the increase of alkaloids in *Datura stramonium* (Berkov and Philipov, 2002) and *Atropa belladonna* (Huang et al., 2010), essential oils and chamazoline in *Matricaria chamomile* (Gosztola et al., 2006), terpenoids and flavonoids in *Salvia miltiorrhiza* (Gao et al., 1996) and morphine content in *Papaver somniferum* (Mishra et al., 2010).

*Calendula* species show a large variation in chromosome numbers. Basic chromosome numbers are 7, 8, 9, 11, and 15 (Rice et al., 2015). *C*. *officinalis* is mostly described as tetraploid, but different chromosome numbers have been published, namely 2*n* = 28 or 2*n* = 32 [Chromosome Counts Database (CCDB) (Rice et al., 2015)]. Higher chromosome numbers are found in *C. arvenisis* (2*n* = 4*x* = 44) and in *C. palaestina* and *C. pachysperma* (2*n* = ± 85), which are probably autopolyploids of *C. arvensis* (Heyn et al., 1974). The differences in chromosome number, karyotype, genome size and ploidy level are the result of high levels of hybridization, chromosome losses and dysploidy (Nora et al., 2013), which makes taxonomy in *Calendula* very difficult. Nora et al. (2013) used genome sizes and chromosome numbers for the evaluation of evolutionary relationships and taxonomy in *Calendula*. They conclude that *C. officinalis* is a tetraploid plant with 32 chromosomes (2*n* = 4*x* = 32) with a genome size of 2.97 ± 0.08 pg/2C.

In our study we aimed to evaluate and confirm the ploidy level and genome size of nine *C*. *officinalis* cultivars using flow cytometry and fluorescent *in situ* hybridization (FISH) analysis. FISH using 45S and 5S rDNA is a valuable method to study plant evolution and ploidy levels (Kirov et al., 2017) and has been used in species such as *Chrysanthemum* (Kondo and Abd El-Twab, 2002; Abd El-Twab and Kondo, 2013), *Alium fistulosum* (Kirov et al., 2017), *Gossypium hirsutum* L. (Andres and Kuraparthy, 2013), *Prunus* species (Schmöllerl, 2009) for the identification of chromosome sets, chromosome numbers, copies and ploidy level. Recently, 45S and 5S rDNA FISH was used to assess the cytogenetic variability of *C. officinalis* after chemical mutagenesis (Samatadze et al., 2019).

The second aim of our study was to perform chromosome doubling in *C*. *officinalis* cultivars establishing a protocol for artificial ploidy doubling. In a seed treatment three antimitotic agents: colchcine, trifluralin and oryzalin, were tested each in three different concentrations in search for an optimal product-dosage combination. First results on differences in cell numbers are given. Next generations of the chromosome doubled plants will be phenotyped with the aim of evaluating them for possible increased yield of biochemical compounds.

## Materials and Methods

### Plant Material

Nine cultivars were used: ‘Orange Beauty,’ ‘Neon,’ ‘Apricot Beauty,’ ‘Cream Beauty,’ ‘Lemon Beauty,’ ‘Yellow Gem,’ ‘Orange Porcupine,’ and ‘Nova’ obtained from Vreeken’s Zaden (Dordrecht, Netherlands) and ‘WUR 1553-7’ obtained from Wageningen University.

### Genome Size of *Calendula* Cultivars

The 2C value of nine *C. officinalis* cultivars (Table 1) was measured using a PASIII flow cytometer (Partec, Germany) equipped with a 20 mW 488 nm laser. The CyStain PI kit (Sysmex, Germany) was used for sample preparation according to the manufacturer’s protocol with minor modifications. One piece (±0.5 cm^2^) of fresh young leaf tissue was chopped together with fresh leaf tissue of the internal standard, *Pisum sativum* ‘Ctirad’ 9.09 pg/2C (Doležel et al., 1998), in 500 μl nuclei extraction buffer (CyStain PI kit). Samples were filtrated through a 50 μl CellTrics filter (Sysmex, Münster, Germany) and subsequently 1200 μl staining solution (CyStain PI kit) with propidium iodide and RNase A stock solution (both CyStain PI kit) were added. Samples were incubated at 4°C for 30 min before measurement. All histograms were analyzed using FloMax software (Quantum Analysis, Germany). For every cultivar two biological replicates were analyzed. The biological replicates were analyzed on different dates and samples were taken from different plants of the same cultivar. For every biological replicate the analysis was repeated (two technical replicates per biological replicate). Mean values and standard deviations were calculated based on the four histograms obtained.

**TABLE 1:** 2C-value (pg) of various *C. officinalis* cultivars.

**Cultivar**	**‘Nova’**	**‘Apricot Beauty’**	**‘Cream Beauty’**	**‘Lemon Beauty’**	**‘Yellow Gem’**	**‘Orange Porcupine’**	**‘Neon’**	**‘Orange Beauty’**	**‘WUR 1553-7’**
2C value (pg)	2.79 ± 0.04	2.76 ± 0.07	2.72 ± 0.04	2.90 ± 0.06	2.76 ± 0.09	2.82 ± 0.05	2.68 ± 0.08	2.80 ± 0.08	2.81 ± 0.06

*Given values represent the mean genome size ± SD of four analyses (two biological replicates with each time a technical replicate).*

### *Calendula* Karyotyping Using 45S and 5S rDNA FISH

Chromosome slides were made for *C. officinalis* ‘Nova,’ ‘WUR 1553-7,’ and ‘Orange Beauty’ according to the ‘SteamDrop’ method (Kirov et al., 2014). Briefly, young root tips were pretreated for 3 h in a solution containing 0.1% colchicine and 8-hydroxyquinoline. Root fixation was done in Carnoy solution 3:1 (ethanol:acetic acid) for 1 h at room temperature. Cell suspensions were made after digestion using 0.6% enzyme solution (mixture of cellulase, pectolyase and cytohelicase) incubation at 37°C for 50 min. During slide preparation 2:1 ethanol:acetic acid and 1:1 ethanol:acetic acid were used as fixative 1 and fixative two, respectively.

Plasmids containing 5S rRNA genes of rye (pSCT7, Lawrence and Appels, 1986) and 45S rRNA genes of wheat (pTA71, Gerlach and Bedbrook, 1979) were labeled by Digoxigenin- and Biotin- Nick Translation Mix (Roche, Germany), respectively, according to the manufacturer’s protocol. For FISH we used the protocol described in Heslop-Harrison et al. (1991) with some modifications. Briefly, slides were incubated overnight at 37°C. Chromosomes were pretreated with 4% paraformaldehyde in 2 × SSC for 8 min at RT and dehydrated in ethanol (70, 90, and 100%). The hybridization mixture consisted of 50% (v/v) deionized formamide, 10% (w/v) dextran sulfate, 2 × SSC, 0.25% sodium dodecyl sulfate, and 2 ng/μl probe DNA. The mixture was denatured at 75°C for 10 min, placed on ice for 5 min, and 80 μl was applied to each slide. Slides were denatured at 80°C for 5 min and incubated overnight at 37°C in a humid chamber. For stringency washing 0.1 × SSC was used at 48°C for 30 min. Biotin and digoxigenin labeled probes were detected with streptavidin-Cy3 (Sigma-Aldrich, United States) and anti-dig-FITC (Roche, Germany), respectively. Slides were counterstained with DAPI [0.2 μl DAPI + 20 μl Vectashield (Labconsult, Brussels, Belgium)] and analyzed using a fluorescence microscope, Zeiss AxioImager M2 (Carl Zeiss MicroImaging, Belgium), equipped with an Axiocam MRm camera and ZEN-software (Carl Zeiss MicroImaging, Belgium). Chromosome and signal analysis was done in DRAWID software version 0.26 (Kirov et al., 2017) on five well-spread metaphases of each cultivar. Chromosome classification and calculation of arm ratios was done according to Levan et al. (1964).

### Chromosome Doubling

Chromosome doubling was performed on *C. officinalis* ‘Nova,’ ‘Orange Beauty,’ and ‘WUR 1553-7’. Seeds were stored under low humidity in a seed storage room at 4°C before use. Dry seeds (about 1000 seeds per cultivar) were primed in 150 ppm GA_3_ for 72 h at room temperature. Per treatment 90 seeds were immersed in different antimitotic agents: 200, 400, 800 ppm colchicine (Duchefa, Netherlands); 20, 40, 80 ppm trifluralin (Sigma-Aldrich, Belgium); 20, 40, 80 ppm oryzalin (Duchefa, Netherlands) and a control treatment using water, during 24 h at room temperature. Stocks of the antimitotic agents were prepared in dimethyl sulfoxide (DMSO, Sigma-Aldrich, Belgium). After treatment the seeds were washed thoroughly with tap water (three times for 5 min). Subsequently for every treatment seeds were germinated on filter paper in Petri dishes. The 90 seeds per treatment were put in three Petri dishes each containing 30 seeds and incubated in the germinator (GC10, Flohr Instruments, Netherlands) with a daylight period of 12 h at 22°C. For the 90 seeds per treatment seed germination was recorded during 14 days. Germinated seeds were transplanted in a plant tray containing a Saniflor peat mixture [(Van Israel, Geraardsbergen, Belgium) 1.5 kg/m^3^; fertilizer: 12N:14P:24 K including trace elements, pH 5.0-6.5, EC 0.450 mS/cm] and placed in the greenhouse (greenhouse conditions: light 16 h in case of shorter day period, day/night: ventilation temperature: 18/22°C, heating temperature 15/18°C etc.). Both germination rate (%) after 14 days and survival rate (%) 3, 6, and 9 weeks after transplanting were calculated.

The ploidy level of the treated seedlings was determined 3, 6, and 9 weeks after transplant using a Cyflow Space flow cytometer (Partec, Germany) equipped with a UV-LED. Plants that were considered chromosome doubled were reanalyzed 6 months later. The protocol used is based on Otto (1990). Approximately 0.5 cm^2^ of young leaf tissue was chopped in a 500 μL extraction buffer containing 0.1 M citric acid monohydrate and 0.5% Tween-20. Samples were filtered through a 50 μm CellTrics filter to eliminate cell debris. Then 750 μL of staining buffer containing 0.4 M Na_2_HPO.12HO, 2 mg/L 4′, 6-diamidino-2-phenyllndole (DAPI), 0.1% polyvinylpyrrolidone (PVP) was added. All histograms were analyzed using FloMax software.

### Cell Number and Size

The cell number ratio between the original and chromosome doubled plants was determined using flow cytometry according to Hias et al. (2017). In the flow cytometric histograms the number of nuclei in the different phases of the cell cycle (G1, S, and G2/M) can be determined using the true volumetric cell counting option of the flow cytometer Cyflow Space (Flomax Software). Sample preparation was done as described above for flow cytometric ploidy analysis. Leaf disks (4 mm diameter) were cut from tetraploid and octaploid plants using a cork borer. The fresh weight (FW) of the leaf disks was determined on a balance (XS104 Mettler-Toledo, Zaventem). In a pooled sample a leaf disk of the tetraploid plant was co-chopped together with a leaf disk of an octaploid plant. Both leaf disks are put on top of each other for chopping. The histogram of the pooled sample with the tetraploid (4x) and octaploid (8x) leaf disks showed three peaks: in the first peak are the G1_4__*x*_ nuclei, a second peaks shows nuclei in G2_4__*x*_ + G1_8__*x*_ (overlapping peaks) and the third peak are the G2_8__*x*_ nuclei. The signal of the nuclei in the S-phase for the tetraploid and octaploid leaf disk is found in between the G1_4__*x*_ and G2_4__*x*_ peaks and G1_8__*x*_ and G2_8__*x*_, respectively. From this histogram the ratio of tetraploid nuclei/g FW over octaploid nuclei/g FW can be determined. To be able to differentiate the overlapping G2_4__*x*_ and G1_8__*x*_ peaks, the percentage of dividing nuclei in the tetraploid leaf is taken into account. Therefore a separate analysis is performed using a leaf disk of the tetraploid plant. From the resulting histogram the percentage of nuclei in division was calculated. A mean ratio was calculated bases on four repetitions.

For histological visualization a modified protocol of Habarugira et al. (2015) was used. Slides were prepared from control and chromosome doubled plant material. Each time leaves and petals were sampled from three different plants of ‘Nova’. Pieces (±1 cm^2^) of full grown leaves and petal pieces (±1 cm length) were harvested and fixated in formalin alcohol acetic acid (FAA) solution (10:7:2:1 EtOH 99%:demineralized water:formalin:glacial acetic acid) in glass tubes and put under vacuum during 20 min. Subsequently the FAA solution on the samples was renewed and stored overnight at 4°C. Samples were rinsed twice with 50% ethanol and preserved at −20°C in 50% ethanol until use. Dehydration was performed by increasing series of ethanol concentrations (70, 85, and 100% during 2 h each time). For the infiltration step 1:1 99% EtOH:glycol methacrylate (Technovit 7100, Heraeus Kuzler, Germany) + 1 g hardener was used. The dehydration was performed in 1.5 ml Eppendorf tubes under vacuum during 20 min. The samples remained for 2 h in the infiltration solution at 4°C. All liquid was removed and replaced by 100% Technovit infiltration solution and kept at 4°C until embedding. For embedding the samples were transferred to 15:1 Technovit:hardener two and kept on ice. The samples were positioned upright in the Eppendorf tube and kept at 4°C for 1 h. Polymerization was performed at 37°C overnight. The samples in the blocks were sectioned at 5 μm using a HM360 Microtome (Thermo Fisher Scientific, Merelbeke, Belgium). Slides were stained for 10 min with 1% Toluidine Blue O (Acros Organics, Geel, Belgium) and rinsed twice with demineralized water. Slides were covered and sealed with DPX mounting medium (Merck KGaA, Darmstadt, Germany). Microscopy was performed using bright field microscopy and pictures were taken (Zeiss AxioImager M2 (Carl Zeiss MicroImaging, Belgium), equipped with an Axiocam MRm camera) and ZEN-software (Carl Zeiss MicroImaging, Belgium).

### Statistical Analysis

The data on seed germination were compared using a one-way ANOVA. If the *F*-test was significant (*p* < 0.05), a Scheffé’s *post hoc* test was performed.

For the data on cell number, a Student’s *t*-test was used. Differences were considered significant when *p* < 0.001.

## Results

### Genome Size and Chromosome Karyotype Analyses on *C. officinalis*

To confirm the genome size and ploidy level of the *C*. *officinalis* cultivars, flow cytometry and karyotype analysis were performed. The mean genome size value (2C) of nine *C. officinalis* cultivars ranged between 2.68 ± 0.08 and 2.90 ± 0.06 pg/2C (Table 1). Since there is only a small variation in *C. officinalis* genome sizes, we can expect that all our *C*. *officinalis* cultivars have the same ploidy level and chromosome count.

**FIGURE 1:**
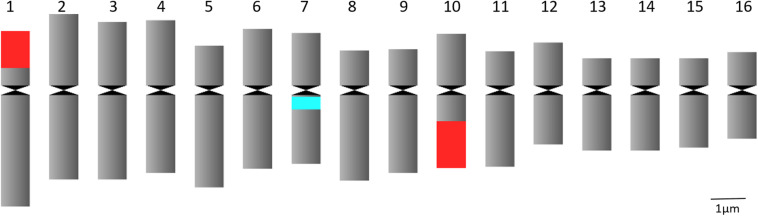
Idiogram of *C. officinalis* chromosomes with indication of 45S rDNA (red) and 5S rDNA (blue) sites.

Chromosome analysis for *C. officinalis* ‘Nova’, ‘WUR 1553-7’ and ‘Orange Beauty’ revealed 32 chromosomes (2*n* = 32). The chromosome length ranges between 1.87 ± 0.37 μm and 3.98 ± 0.53 μm (Figure 1 and Table 2). The karyotype contains two sets of 16 homoeologous chromosomes showing a karyotype formula 2(8M + 8SM), with chromosomes 2, 3, 4, 6, 7, 10, 12, and 16 metacentric (M) chromosomes while the rest were submetacentric (SM) (Table 2 and Figure 1).

**TABLE 2:** Chromosome characteristics of *Calendula officinalis.*

**Chromosome name**	**Centromere index (%)^a^ ± SD**	**Chromosome length (μm) ± SD**	**Short arm length (μm) ± SD**	**Long arm length (μm) ± SD**	**Arm ratio**	**Chromosome designation^b^**
1	32.03 ± 4.02	3.99 ± 0.53	1.26 ± 0.39	2.72 ± 0.31	2.38 ± 0.88	SM
2	46.02 ± 3.30	3.82 ± 0.56	1.75 ± 0.27	2.06 ± 0.35	1.18 ± 0.17	M
3	42.68 ± 3.85	3.57 ± 0.65	1.52 ± 0.29	2.05 ± 0.42	1.26 ± 0.36	M
4	45.62 ± 3.47	3.48 ± 0.74	1.59 ± 0.36	1.89 ± 0.42	1.21 ± 0.19	M
5	29.83 ± 3.98	3.21 ± 0.54	0.94 ± 0.25	2.26 ± 0.40	2.57 ± 0.41	SM
6	43.33 ± 2.93	3.16 ± 0.52	1.36 ± 0.22	1.79 ± 0.34	1.30 ± 0.16	M
7	43.3. ± 3.86	2.96 ± 0.47	1.29 ± 0.26	1.68 ± 0.26	1.32 ± 0.21	M
8	29.14 ± 3.16	2.93 ± 0.51	0.85 ± 0.13	2.09 ± 0.42	2.47 ± 0.40	SM
9	31.45 ± 2.65	2.77 ± 0.26	0.87 ± 0.11	1.90 ± 0.19	2.20 ± 0.31	SM
10	45.24 ± 3.55	2.77 ± 0.53	1.21 ± 0.23	1.56 ± 0.36	1.30 ± 0.25	M
11	31.80 ± 2.73	2.58 ± 0.35	0.82 ± 0.13	1.76 ± 0.25	2.17 ± 0.31	SM
12	46.01 ± 3.30	2.24 ± 0.51	1.04 ± 0.26	1.21 ± 0.26	1.18 ± 0.16	M
13	32.99 ± 4.44	2.03 ± 0.46	0.67 ± 0.17	1.37 ± 0.33	2.09 ± 0.47	SM
14	32.80 ± 3.00	2.00 ± 0.28	0.65 ± 0.11	1.34 ± 0.20	2.07 ± 0.29	SM
15	34.05 ± 3.00	1.95 ± 0.39	0.66 ± 0.14	1.29 ± 0.27	1.96 ± 0.27	SM
16	43.23 ± 4.76	1.87 ± 0.37	0.75 ± 0.27	1.12 ± 0.18	1.72 ± 0.91	M

*^*a*^Values are means (*n* = 4) ± SD; ^*b*^M, Metacentric and SM, Sub-metacentric.*

**FIGURE 2:**
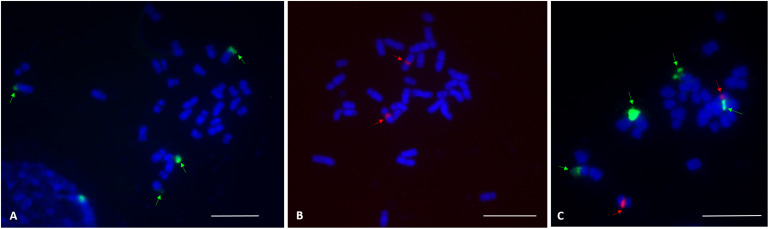
FISH localization of 45S rDNA and 5S rDNA loci (indicated by arrows) on mitotic metaphase chromosomes of *C. officinalis* ‘Nova.’ **(A)**, four 45S rDNA signals were identified in the terminal regions of chromosome pair 1 and 10. **(B)**, Two 5S rDNA signals were detected in the centromeric region of chromosome pair 7. **(C)**, 45S rDNA (green) and 5S rDNA (red) signals. Bar = 10 μm.

FISH analysis with the 45S rDNA probe revealed four signals located at the terminal region of the chromosome pairs 1 and 10 (Figures 2A,C). Two bright signals of the 5S rDNA probe hybridization were observed in the pericentromere region of chromosome pair 7 (Figures 2B,C). Multicolor FISH analysis using both 45S and 5S rDNA probes confirmed the four 45S rDNA and two 5S rDNA signals (Figure 2C). The signals were observed in all analyzed metaphase cells. The karyotype indicates an allotetraploid background in *C. officinalis*.

### Polyploidization in *C*. *officinalis*

#### Seed Germination After Polyploidization

Chromosome doubling using antimitotic agents was assessed on seeds of three allotetraploid *C. officinalis* cultivars with the aim to obtain octaploids. The effect of the antimitotic agents on seed germination is shown in Table 3. Seed germination was affected by the use of antimitotic agents in all three cultivars, as shown by the one-way ANOVA [‘Nova’ *F* = 9.12, *p* = 0.00002; ‘Orange Beauty’ *F* = 4.79, *p* = 0.0017; ‘WUR 1553-7’ *F* = 2.93, *p* = 0.026]. Although in all three cultivars and for every treatment a lower number of seeds germinated when compared to the control, the Scheffé’s *post hoc* test showed that the negative effect of the application of colchicine is only significant at the higher doses of 400 and 800 ppm, and not at 200 ppm. The negative impact on seed germination was more pronounced when higher concentrations of colchicine were applied. When compared to the control treatment, seed germination in ‘Nova’ significantly decreased for all oryzalin and trifluralin concentrations applied, but for ‘Orange Beauty’ and ‘WUR 1553-7’ the decrease in seed germination is less severe and differs significantly in only a few treatments when compared to the control treatment. For oryzalin and trifluralin no dose response is observed as no significant differences are found between concentrations used. However, high concentrations of antimitotic agents resulted in a delay in root emergence and abnormal seedlings were observed that had compact roots without a root tip and with fragile cotyledons.

**TABLE 3:** Seed germination for three *C. officinalis* cultivars after treatment with antimitotic agent treatments in different concentrations.

**Antimitotic agent**	**Concentration (ppm)**	**% Seed Germination**		
		**‘Nova’^∗^**	**‘Orange Beauty’**	**‘WUR 1553-7’**
		**% ± SD**	**% ± SD**	**% ± SD**
Control	0	73.3 ± 3.8^*a*^	85.3 ± 1.9^*a,b*^	81.3 ± 12.4^*a*^
Colchicine	200	42.2 ± 12.3^*a,b*^	68.9 ± 7.9^*a,b*^	63.3 ± 7.2^*a,b*^
	400	25.6 ± 11.3^*b*^	66.7 ± 11.9^*a,b,c*^	46.7 ± 4.7^*a,b*^
	800	8.9 ± 4.7^*b*^	45.6 ± 4.2^*b,c*^	40.0 ± 9.4^*b*^
Oryzalin	20	18.9 ± 6.3^*b*^	60.0 ± 2.7^*a,b,c*^	44.4 ± 3.1^*a,b*^
	40	15.6 ± 5.7^*b*^	61.1 ± 12.3^*a,b,c*^	40.0 ± 5.4^*b*^
	80	15.6 ± 1.6^*b*^	46.7 ± 9.4^*b,c*^	48.9 ± 15.0^*a,b*^
Trifluralin	20	15.6 ± 4.3^*b*^	60.0 ± 9.8^*a,b,c*^	57.8 ± 5.7^*a,b*^
	40	27.8 ± 8.7^*b*^	32.2 ± 3.1^*c*^	46.7 ± 5.4^*a,b*^
	80	14.4 ± 8.3^*b*^	58.9 ± 6.3^*a,b,c*^	48.9 ± 4.2^*a,b*^

*For each treatment mean values (%) and standard deviations (±SD) are calculated based on the three Petri dishes with 30 seeds in each Petri dish. ^∗^within one genotype different letters exhibit statistically significant differences (Scheffé’s test; *p* < 0.05).*

### Ploidy Analysis

For all three cultivars antimitotic agents had a negative effect on seedling survival. Nevertheless octaploids and mixoploids were obtained for all three antimitotic agents, but the efficiency depended on the *Calendula* genotype (Tables 4–6). A high rate of reversion to the tetraploid level was observed. In total 168 ‘Nova’ seedlings were transplanted after treatment. The highest seedling mortality was observed between 3 and 6 weeks after transplant when only 23 seedlings were still alive. After 9 weeks 21 seedlings survived. In ‘Nova’ the highest number of stable tetraploid plants was obtained using 200 ppm colchicine and oryzalin or trifluralin at a concentration of 20 ppm (Table 4). For ‘Orange Beauty’ the number of surviving seedlings also dropped most between 3 and 6 weeks after transplanting, from 455 transplanted to 313 after 3 weeks and 43 seedlings after 6 weeks (Table 5). Three weeks after transplanting, 32 octaploid plants were found, while only 2 octaploid seedlings were left after 6 weeks and only one after 9 weeks. A high number of mixoploid seedlings (75 seedlings) was detected 3 weeks after transplanting, but again after 9 weeks, the number decreased to only 10 mixoploid seedlings (Table 5). Most mixoploids either died or reverted back to the tetraploid level. Also in the other cultivars it is observed that the number of tetraploids increases over time because octaploids or mixoploids return to the tetraploid level. After germination, 393 ‘WUR 1553-7’ seedlings were transplanted (Table 6). For this cultivar as well, most of the seedlings died between 3 and 6 weeks after transplant. In total 37 octaploid plants were detected 3 weeks after transplant and only 16% of them (6 seedlings) remained octaploid after 9 weeks. Most octaploid plants were obtained after colchicine treatments and 80 ppm oryzalin (Table 6). The ploidy analysis on the chromosome doubled plant 6 months later confirmed the stability of these octaploids.

**TABLE 4:** The number of surviving seedlings and ploidy level of ‘Nova’ treated with various antimitotic agents.

**‘Nova’**		**Number of transplanted seedlings**	**Number of surviving seedlings**			**Tetraploid plants**			**Octaploid plants**			**Mixoploid plants**		

**Antimitotic agent**	**Conc. (ppm)**		**Weeks after transplanting**											
			**3**	**6**	**9**	**3**	**6**	**9**	**3**	**6**	**9**	**3**	**6**	**9**
Colchicine	200	38	33	6	5	26	3	3	4	3	2	3	0	0
	400	22	11	3	3	9	1	3	1	1	0	1	1	0
	800	9	5	3	3	4	0	2	1	1	1	0	2	0
Oryzalin	20	17	16	2	2	15	1	1	1	1	1	0	0	0
	40	14	8	0	0	5	0	0	0	0	0	3	0	0
	80	14	5	2	2	1	0	1	1	1	0	3	1	1
Trifluralin	20	16	16	2	2	14	1	1	1	1	1	1	0	0
	40	25	10	1	1	8	1	1	1	0	0	1	0	0
	80	13	11	4	3	7	2	2	0	0	0	4	2	1
Total		168	115	23	21	89	9	14	10	8	5	16	6	2

*Each treatment contains three Petri dishes with 30 seeds in each Petri dish. Numbers are given on the total of 90 seeds per treatment.*

**TABLE 5:** The number of surviving seedlings and ploidy level of ‘Orange Beauty’ treated with various antimitotic agents.

**‘Orange Beauty’**		**Number of transplanted seedlings**	**Number of surviving seedlings**			**Tetraploid plants**			**Octaploid plants**			**Mixoploid plants**		

**Antimitotic agent**	**Conc. (ppm)**		**Weeks after transplanting**											
			**3**	**6**	**9**	**3**	**6**	**9**	**3**	**6**	**9**	**3**	**6**	**9**
Colchicine	200	62	34	3	3	26	2	2	1	0	0	7	1	1
	400	60	29	5	5	14	1	2	2	1	1	13	2	2
	800	41	9	2	2	3	0	0	1	1	0	5	1	2
Oryzalin	20	54	49	7	7	36	6	5	4	0	0	9	1	2
	40	55	50	2	2	30	1	1	1	0	0	19	1	1
	80	42	13	7	7	3	2	6	3	0	0	7	5	1
Trifluralin	20	59	59	1	1	45	1	1	9	0	0	5	0	0
	40	29	23	7	7	18	6	7	3	0	0	2	1	0
	80	53	47	9	9	31	7	8	8	0	0	8	2	1
Total		455	313	43	43	206	26	32	32	2	1	75	14	10

*Each treatment contains three Petri dishes with 30 seeds in each Petri dish. Numbers are given on the total of 90 seeds per treatment.*

**TABLE 6:** The number of surviving seedlings and ploidy level of ‘WUR 1553-7’ treated with various antimitotic agents.

**‘WUR 1553-7’**		**Number of transplanted seedlings**	**Number of surviving seedlings**			**Tetraploid plants**			**Octaploid plants**			**Mixoploid plants**		

**Antimitotic agent**	**Conc. (ppm)**		**Weeks after transplanting**											
			**3**	**6**	**9**	**3**	**6**	**9**	**3**	**6**	**9**	**3**	**6**	**9**
Colchicine	200	57	44	3	3	37	1	2	7	0	1	0	2	0
	400	42	29	8	8	15	2	3	10	1	2	4	5	3
	800	36	10	4	4	5	0	0	2	1	1	3	3	3
Oryzalin	20	40	36	7	5	32	3	4	2	0	0	2	4	1
	40	36	36	5	5	26	2	5	8	0	0	2	2	0
	80	44	21	10	9	12	2	4	3	3	2	6	5	3
Trifluralin	20	52	49	0	0	48	0	0	1	0	0	0	0	1
	40	42	30	4	4	22	4	3	4	0	0	4	0	1
	80	44	21	21	21	19	16	15	0	0	0	2	5	6
Total		393	276	62	59	216	30	36	37	5	6	23	26	18

*Each treatment contains three Petri dishes with 30 seeds in each Petri dish. Numbers are given on the total of 90 seeds per treatment.*

**FIGURE 3:**
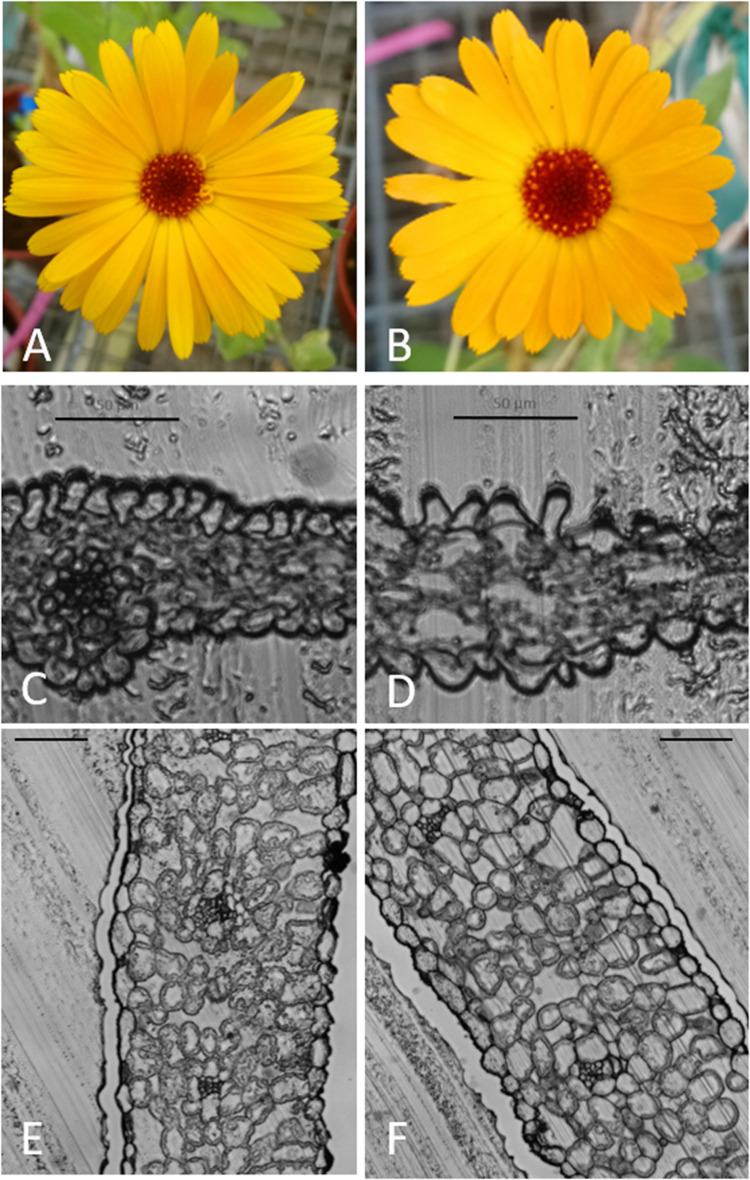
Flowering in tetraploid and octaploid *Calendula officinalis* ‘Nova’ and cross sections of leaf and petal. **(A,C,E)**: flower, flower cross section and leaf cross section of a control plant. **(B,D,F)**: flower, flower cross section and leaf cross section of a chromosome doubled plant. **(C,D)** bar = 50 μm; **(E,F)** bar = 100 μm.

For all three cultivars, octaploid plants exhibited a slower growth rate compared to tetraploid control plants in the first weeks after transplantation. However, after 3 weeks the remaining octaploid plants showed increased growth vigor compared to the tetraploid control plants. Flowering was observed in both the tetraploid and the octaploid plants (Figures 3A,B).

### Cell Number

A significant difference (*t*-test; *p* < 0.001) was found when the weight of the 4 mm diameter punches of the control and the chromosome doubled leaves were compared. The weight of the control leaf disks was 4.4 ± 0.2 mg while it was 5.8 ± 0.6 mg for the chromosome doubled leaf disks.

When the number of nuclei in the original and the chromosome doubled leaf disks was calculated, the number of nuclei per gram is significantly lower in the chromosome doubled plants (*p* < 0.001; *t*-test). When the ratio is calculated for every combined analysis of undoubled and chromosome doubled leaf disks, an average of 3.16 ± 0.91 times more cells were observed in the non-doubled plants.

Leaves and flowers of the chromosome doubled and control plants were prepared as slides. Microscopic images confirmed that the leaves and flowers of chromosome doubled leaves were thicker and both flowers and leaves had bigger cells (Figures 3C–F).

## Discussion

Despite the number of studies published on the evolution in the genus *Calendula*, some ambiguity remains about chromosome numbers and ploidy levels. In our study, genome sizes between 2.68 ± 0.08 and 2.90 ± 0.05 pg/2C were found for the nine *C. officinalis* cultivars tested, which is a bit lower than the 2.97 ± 0.08 pg mentioned in literature (Nora et al., 2013). Intraspecific variations of 2C values in plants is subject to controversy. It is difficult to separate intraspecific variation in genome size caused by cytolic compounds from various molecular mechanisms including duplications, deletions, chromosomal polymorphisms, the presence of B-chromosomes and/or the presence of repetitive sequences. For example Bilinski et al. (2018) observed differences in genome sizes in maize landraces correlated to abundance of transposable element families and heterochromatic knobs. We observed small but significant differences in genome sizes in *C. officinalis* cultivars between greenhouse grown plants and field grown plants (results not shown). Therefore we assume that the variation in genome sizes between cultivars of *C. officinalis* is due to stoichiometric errors of the flow cytometric analysis and by variations in levels of secondary metabolites (Greilhuber, 1998; Noirot et al., 2000; Huang et al., 2013).

Infra-generic variation in genome size among homoploid species is a common feature in plants (Bennett and Leitch, 2011). In *Calendula* taxa a positive correlation is found between genome size and chromosome number (Nora et al., 2013). But for *C. officinalis* the genome size is lower than expected when compared to other *Calendula* species with the same chromosome number. Evaluating different possibilities of interspecific hybridizations and ploidy doubling, Nora et al. (2013) assumed, based on genome sizes, that dysploidy might been involved in the origin of *C. officinalis*. For *C. officinalis* either 28 or 32 chromosomes are mentioned, depending on the author (Rice et al., 2015). Garcia et al. (2010) reported 28 chromosomes while a recent study (Samatadze et al., 2019) reported 32 chromosomes. In the present study, chromosomal slides were prepared and an ideogram was made based on the counted number of 32 chromosomes. In addition, Garcia et al. (2010) observed four 45S rDNA and two 5S rDNA signals after FISH analysis. This was confirmed by Samatadze et al. (2019) who describe four signals of 45S rDNA on chromosome pairs 1, 9, weak (polymorphic) 45S rDNA signals on chromosome 5 and 10 and 2 signals of 5S rDNA on chromosome pair 10. In our study, we also observed four signals for 45S rDNA on chromosome pair number 1 and 10 and 2 signals for 5S rDNA on chromosome pair 7. The differences in chromosome identification between our study and the study of Samatadze et al. (2019) are most probably due to the small and rather uniform chromosomes of *C. officinalis*. When taken together, the following observations strongly indicate that *C. officinalis* is an allotetraploid species: (1) the prevalence of four signals of 45S rDNA on two different chromosome pairs and two signals of 5S rDNA on one chromosome pair taken together with the number of chromosomes, (2) the morphology of the chromosomes illustrating the fact that chromosomes morphologically appear in sets of two. Autopolyploids arise from intra-species whole genome duplication events, while allopolyploids arise from genome duplication events involving inter-specific hybridization (Ramsey and Schemske, 2002). Nora et al. (2013) discuss different hypotheses to explain the chromosome number and the genome size in *C. officinalis*. Both hypotheses, chromosome losses in *C. maroccana* (Heyn and Joel, 1983) or hybridization between *C. stellata* (2*n* = 14) and a species with 2*n* = 18 (e.g., C. *maroccana*, *C. eckerleinii*) followed by chromosome doubling (Ohle, 1974) are not well supported when the genome sizes of these species are taken into account. Other techniques like genomic *in situ* hybridization (GISH) using probes of the possible ancestors could be used to explore what species are involved in the genetic background in *C. officinalis* as well as to examine the loss of specific chromosomes. Further, intra-genomic and inter-genomic meiotic pairings can be studied in allo- and autopolyploids to discriminate homologous and homoeologous chromosomes (Lloyd and Bomblies, 2016); this could be interesting to study introgression breeding.

The indications for allopolyploidy in *C. officinalis* and the higher chromosome numbers found in some other *Calendula* species (e.g., 2*n* = ± 85 in *C. palaestina* and *C. pachysperma*) (Heyn et al., 1974) led us to explore chromosome doubling in *C. officinalis*. Genome doubling is often induced by treating seeds with antimitotic agents (Salma et al., 2017). Colchicine is the most common substance for chromosome doubling in plants, but it has several undesirable side effects: it is highly toxic to humans, shows high sensitivity to light, and shows low affinity to plant tubulin in low concentrations (Dhooghe et al., 2010; Eeckhaut et al., 2018; Eng and Ho, 2019; Manzoor et al., 2019). We therefore also included oryzalin and trifluralin in our study. Due to their better affinity for plant tubulin, lower concentrations (one-tenth of colchicine) can be used (Dhooghe et al., 2010; Sattler et al., 2016; Eeckhaut et al., 2018).

A general trend observed in the present study was that as the metaphase inhibitor concentrations increased, seed germination decreased. Among our *C. officinalis* cultivars, ‘Orange Beauty’ had the highest seed germination compared. Lowest germination rates were found in ‘Nova’, both as control and in the treatments. Especially for colchicine, higher doses resulted in lower seed germination rates. For oryzalin and trifluralin the decrease in germination was less pronounced and the results were more variable. Our results were in agreement with previous studies that showed a fatal effect of high doses of antimitotic agents on seeds (Luo et al., 2018; Nasirvand et al., 2018). High doses of these substances are toxic to plant cells and modify various plant activities (Pintos et al., 2007; Luo et al., 2018). Moreover, the antimitotic agents were dissolved in DMSO and exposure lasted relatively long (24 h). DMSO increases cell wall permeability to antimitotic agents and thereby augments effectiveness (Tavan et al., 2015; Eng and Ho, 2019). On the other hand, in high concentrations DMSO is lethal to cells and can lead to a reduction of the root tip mitotic index (Dillé and King, 1983). Deleterious effects of antimitotic agents have been reported in various plants, not only in terms of seed germination but also in treated explants throughout the life cycle (Eng and Ho, 2019; Manzoor et al., 2019). In our study, abnormal seedlings with small and thick roots without root tips and hairs were obtained (especially after high dosages), and these seedlings died after transplant. The same was observed in *Taraxacum* (Luo et al., 2018) and *Onobrychis elata* (Avci et al., 2019). In addition, the octaploid *C. officinalis* plants grew slower than tetraploid seedlings during the first weeks. In this study ploidy levels were estimated using flow cytometry, therefore the occurrence of aneuploidy cannot be excluded and should be checked by chromosome counts in root metaphases after seed propagation.

Analysis of putative polyploid seedlings showed that doses of 200 and 400 ppm of colchicine were most efficient for chromosome doubling in ‘Nova’ and ‘Orange Beauty’, respectively, while treatment with 80 ppm oryzalin was also effective for ‘WUR 1553-7’. However, the very low number of octaploids makes it difficult to make general conclusions. Other studies have shown that the optimal antimitotic agent and concentration is strongly genotype-dependent (Dhooghe et al., 2010), such as in parsley (Nasirvand et al., 2018), *Onobrychis elata* (Avci et al., 2019), and *Escallonia* (Denaeghel et al., 2018), among many others. Unfortunately most of the octaploid plants detected at 3 weeks after transplant reverted to tetraploid status after 9 weeks. Unstable polyploidization has been observed in other plants, such as *Eriobotrya japonica* (Blasco et al., 2015), *Gerbera jamesonii* (Gantait et al., 2011), etc. Probably reversion is due to the higher mitotic index in tetraploids versus their octaploid counterparts whereby the tetraploid cells multiply faster. Application of oryzalin and trifluralin was less effective due to the low survival rate, especially in ‘Nova.’ Colchicine is applied in higher concentrations when compared to oryzalin and trifluralin because of its lower affinity for plant tubulin.

Generally, polyploidy is associated with a higher leaf size, thicker leaves and stem diameter, shorter internodes and superior agronomic traits (‘giga’ effects) as compared to their diploid counterparts (Li et al., 2018; Luo et al., 2018; Eng and Ho, 2019). These changes caused by polyploidization led to the tendency of plant breeders to test chromosome doubling as an efficient breeding tool (Sattler et al., 2016; Corneillie et al., 2019). However, the increase of plant and organ size is not correlated to the polyploidy level. It is known that ploidy has an optimum level. Tetraploids are often bigger than triploids, which are in turn bigger than diploids. But it is also possible that tetraploids have a more compact growth habit as compared to diploids, as shown in *Escallonia* (Denaeghel et al., 2018). Higher ploidy levels are often marked by stunted growth as was shown in *A. thaliana* (Corneillie et al., 2019). Therefore the effect of polyploidization cannot be predicted. In most research on induced polyploidy, morphological characteristics are studied, but usually not with a micro-morphological approach. In our study, the number of leaf cells was shown to decrease in octaploid plants; however, the leaves of the octaploids have a higher weight. Hias et al. (2017) also made this observation after chromosome doubling in diploid apple, where the number of nuclei per leaf area decreased by a factor of ±2,42 after chromosome doubling. We can assume that for *C. officinalis* this decrease in cell number is associated with an increase in cell size. Indeed, first observations in the leaf cross-sections show that leaves are thicker and cell size increases in octaploid plants. This phenomenon has been reported also in other polyploid plants, such as (4x, 6x, and 8x) *Arabidopsis thaliana* (Corneillie et al., 2019), tetraploid plants of *Juncus effucus* (Xu et al., 2010), and *Limonium bellidifolium* (Mori et al., 2016). This affects plant growth and production of secondary metabolites. Therefore we will perform a more thorough study growing the offspring of the plants with different ploidy levels in bigger numbers and under crop-appropriate field conditions to phenotype them.

## Conclusion

*C. officinalis* probably has an allotetraploid background. Its economic importance and the occurrence of other *Calendula* species with higher chromosome numbers makes *C*. *officinalis* an interesting candidate for chromosome doubling. High dosages of antimitotic agents have a toxic effect on seed germination and survival of seedlings. Application of colchicine in low concentrations (about 200–400 ppm) is the most efficient antimitotic agent for chromosome doubling in *Calendula*. The number of octaploids obtained is low (maximum 2.2%), but the plants obtained are valuable for further breeding as well as the study of the agricultural value and metabolite profiles among ploidy levels. Such insights can lead to development of better performing cultivars.

## Data Availability Statement

The datasets generated for this study are available on request to the corresponding author.

## Author Contributions

GE conducted the experimental work in cytogenetics, polyploidization, and flow cytometry. KV contributed to the experimental work on cytogentics. HM contributed to the plant experimental work. LL contributed to the experimental work on polyploidization and flow cytometry. GE, KV, HM, and LL participated in the writing of the manuscript.

## Conflict of Interest

The authors declare that the research was conducted in the absence of any commercial or financial relationships that could be construed as a potential conflict of interest.
